# Energy Metabolism on Mitochondrial Maturation and Its Effects on Cardiomyocyte Cell Fate

**DOI:** 10.3389/fcell.2022.886393

**Published:** 2022-07-05

**Authors:** Kaya L. Persad, Gary D. Lopaschuk

**Affiliations:** Department of Pediatrics, Cardiovascular Research Centre, University of Alberta, Edmonton, AB, Canada

**Keywords:** mitochondrial maturation, metabolism, cell fate, postnatal development, cardiomyocyte, glycolysis mitochondrial contribution to cell maturation

## Abstract

Alterations in energy metabolism play a major role in the lineage of cardiomyocytes, such as the dramatic changes that occur in the transition from neonate to newborn. As cardiomyocytes mature, they shift from a primarily glycolytic state to a mitochondrial oxidative metabolic state. Metabolic intermediates and metabolites may have epigenetic and transcriptional roles in controlling cell fate by increasing mitochondrial biogenesis. In the maturing cardiomyocyte, such as in the postnatal heart, fatty acid oxidation increases in conjunction with increased mitochondrial biogenesis driven by the transcriptional coregulator PGC1-α. PGC1-α is necessary for mitochondrial biogenesis in the heart at birth, with deficiencies leading to postnatal cardiomyopathy. While stem cell therapy as a treatment for heart failure requires further investigation, studies suggest that adult stem cells may secrete cardioprotective factors which may regulate cardiomyocyte differentiation and survival. This review will discuss how metabolism influences mitochondrial biogenesis and how mitochondrial biogenesis influences cell fate, particularly in the context of the developing cardiomyocyte. The implications of energy metabolism on stem cell differentiation into cardiomyocytes and how this may be utilized as a therapy against heart failure and cardiovascular disease will also be discussed.

## Introduction

Heart failure affects over 50 million people worldwide, and as such has serious health and economic impacts on our society (Cowie et al., 1997; McMurray and Stewart, 2002; Braunwald, 2015). One reason for this burden is that it is the most common reason for hospitalization, particularly in older individuals (Braunwald, 2015). Given its widespread effects on both individual health and global health care, it is important to find adequate treatments. Currently, there are several pharmacological and surgical therapies that have been developed to treat heart failure (reviewed respectively by Egbuche et al., 2020 and Allen and Felker, 2008). Two potential additional approaches to treat heart failure are to prevent further cardiomyocyte cell death, or to actually regenerate injured cardiac muscle. The adult cardiomyocyte does not proliferate; therefore, the use of stem cells for regenerating cardiomyocytes is a potential approach to treat heart failure (Heallen et al., 2019). Properly functioning mitochondria are critical to regulating cardiomyocyte cell death (reviewed in Orogo and Gustafsson, 2013) and cardiomyocyte maturation (reviewed in Garbern and Lee, 2021). Therefore, a better understanding of the role of mitochondria in determining cell fate is important. Of note, there are significant changes in mitochondrial energy metabolism in the failing heart (reviewed in Lopaschuk et al., 2010). This includes a loss of metabolic flexibility that can lead to a reduction in ATP production (Lopaschuk et al., 2021). This is likely due to the reduced mitochondrial oxidative capacity that is experienced during heart failure, with the heart reverting to a more fetal like state. In the fetal cardiomyocyte, the main source of energy production is through the glycolytic pathway, which is associated with a higher proliferative capacity (Lopaschuk et al., 1991).

Cardiomyocyte maturation in the developing heart can be characterized by their ability to contract forcefully and in a synchronized manner. As reviewed in Momtahan et al. (2019), mature postnatal cardiomyocytes are phenotypically more elongated, have organized sarcomeres and see a 30-to-40-fold increase in cardiomyocyte size. In order to support the rapid and forceful contractions, mature cardiomyocyte have well-developed sarcoplasmic reticulum and transverse-tubules, which allows for calcium-induced calcium release (Momtahan et al., 2019). When cardiomyocytes are regenerated through stem cells, they typically maintain more immature characteristics, although multiple methods have been attempted to increase their maturation (reviewed in Karbassi et al., 2020). Notably, no method has been able to mature regenerated cardiomyocytes to the same extent as primary adult cardiomyocytes (Karbassi et al., 2020). Adding to this issue, a number of cardiac pathologies can result in a delayed or decreased maturation of the cardiomyocyte. For instance, hypertrophic cardiomyopathy can occur in adolescent patients, and presents with a hypertrophied left ventricle (Reviewed in Soler et al., 2018). Diastolic dysfunction is an early hallmark of hypertrophic cardiomyopathy, and the cardiomyocytes can revert to a less mature more fetal-like state (Fukushima et al., 2018; Soler et al., 2018).

Given the significant changes in energy metabolism seen in both the developing and failing heart, a better understanding of what changes in mitochondrial dynamics, homeostasis, and energy metabolism occur in these hearts is important. Valuable insights into the role of mitochondria in determining cell fate can also be obtained from studies involving other proliferating cells such as cancerous cells and stem cells. Therefore, this review discusses the potential implications of alterations in mitochondrial dynamics, homeostasis, and energy metabolism in determining cell fate not only in cardiomyocytes but also in stem cell and cancerous cells.

## Mitochondrial Dynamics and Homeostasis and Its Role in Determining Cell Fate

**FIGURE 1:**
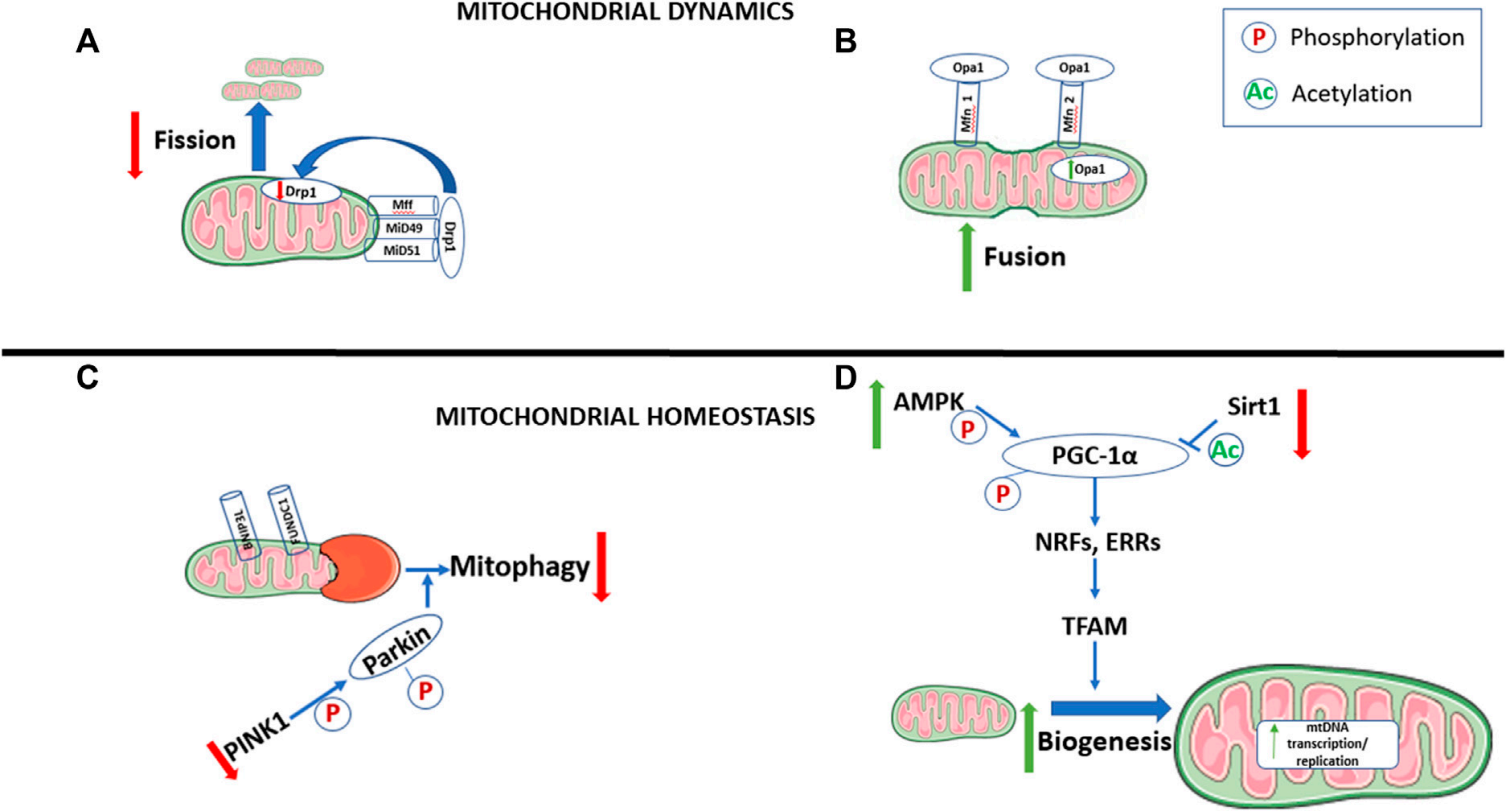
Changes in mitochondrial dynamics and homeostasis during cell maturation. During the maturation of cells that are transitioning from a proliferating to differentiated state, there is an increase in mitochondrial fusion **(A)** and biogenesis **(D)**, with decreases in mitophagy **(C)** and mitochondrial fission **(B)**. Pathways involved in mitophagy, and fission are also downregulated, while pathways promoting mitochondrial fusion and biogenesis are upregulated. AMP-activated protein kinase (AMPK). BCL2 interacting protein 3 like (BNIP3L). Dynamin-related protein 1 (Drp1). Estrogen-related receptors (ERRs). FUN14 domain-containing-1 (FUNDC1). Mitochondrial fission factor (Mff). Mitofusin 1/2 (Mfn 1/2). Mitochondrial dynamic protein 49/51 (MiD49/51). Mitochondrial DNA (mtDNA). Nuclear respiratory factors (NRFs). Optic atrophy 1 (Opa1). Peroxisome proliferator-activated receptor-γ coactivator-1α (PGC-1α). Phosphatases and tensin homolog (PTEN)-induced putative kinase 1 (PINK1). Sirtuin 1 (Sirt1). Mitochondrial transcription factor A (TFAM).

The mitochondrion is an organelle that is the “powerhouse” of the cell, as it hosts a multitude of metabolic processes. Most notably, it produces energy in the form of adenosine triphosphate (ATP) through oxidative phosphorylation. The mitochondria within our cells are constantly changing to adapt to changes in the microenvironment of the cell and the stress that the cells experience. Changes in mitochondrial characteristics also have an important role in determining cell fate (Figure 1). These changes are facilitated through fission and fusion, which manipulate mitochondrial number, size, and orientation within the cell, also known as mitochondrial dynamics (Liesa et al., 2009), as well as mitophagy, which controls mitochondrial quality (Lemasters, 2005) and mitochondrial biogenesis, which controls mitochondrial quantity and size, both of which are categorized under mitochondrial homeostasis (Palikaras and Tavernarakis, 2014). These processes are regulated by large guanosine triphosphatases (GTPases) in the dynamin family of proteins (Hoppins et al., 2007). Mitochondrial dynamics is key to determining the morphology and ultimately the energy metabolic capacity of the cell. Fission and fusion also allow for mitochondria to exchange metabolites, proteins, and mitochondrial DNA (mtDNA) which keeps mitochondria healthy and dilutes any potential abnormalities (reviewed in Abel, 2018; Mishra and Chan, 2016). As such, the effect that mitochondrial dynamics imposes on cell fate is critical. Importantly, mitochondrial dynamics also play a significant role in the fate of cardiomyocytes (Nikoletopoulou et al., 2013; Rambold and Pearce, 2018).

### Fission and Fusion

Fission is the process in which one mitochondrion divides into two mitochondria. As such, mitochondrial fission can facilitate growth and proliferation in the cell by generating enough mitochondria for two independent cells (Chen et al., 2018; Zhan et al., 2016). The key protein involved in mediating fission in mammals is dynamin-related protein 1 (Drp1), a cytosolic dynamin family member. Drp1 is recruited to the outer mitochondrial membrane (OMM) of the mitochondrion through receptors, mitochondrial fission 1 protein (Fis1), mitochondrial dynamics protein 49 and 51 (MiD49 MiD51, respectively), and mitochondrial fission factor (Mff) (Elgass et al., 2013) (Figure 1). Phosphorylation of Fis1 promotes mitochondrial fission through increased recruitment of Drp1 (Yu et al., 2021; Wang et al., 2022). Once Drp1 is recruited to the mitochondria, it oligomerizes and facilitates membrane constriction in a GTP-dependent manner (Kraus and Ryan, 2017). Drp1 is translocated to pre-constricted mitochondrion-endoplasmic reticulum (ER) contact cites, which promotes further Drp1 recruitment and mitochondrial fission (Ortiz-Sandoval et al., 2014). Indeed, Mff-dependent Drp1 recruitment is initiated when the ER begins to surround the mitochondrion proceeding with immediate mitochondrial division (Rowland and Voeltz, 2012). Post-translation modifications of Drp1, such as phosphorylation, have been previously reviewed (Hu et al., 2017) and whether or not it leads to the activation or inactivation of Drp1 is largely dependent on the stimuli and the residue site. Recruitment of Drp1 into the outer mitochondrial membrane and the subsequent narrowing of the membrane lead to mitochondrial fragmentation and division (Smirnova et al., 2001; Fröhlich et al., 2013).

During the postnatal development of the heart, mitochondrial fission is essential in diffusing nucleoids within the cardiomyocyte, allowing for a normal distribution of activate mitochondria (Ishihara et al., 2015). This is important, as insufficient distribution within the cytoplasm can impede cardiomyocyte growth due to a lack of adequate energy supplies. In fact, Ishihara et al. (2015) observed a cardiac-specific respiration deficiency in Drp1-ablated hearts due to impaired assembly of the respiratory complex. Deletion of Drp1 in the postnatal cardiomyocyte leads to abnormal mitochondrial morphology and fatal cardiomyopathy with the first 1–2 weeks of life (Kageyama et al., 2014; Ishihara et al., 2015). Mitochondrial fission is also an important process that occurs in the adult heart, although studies are not as consistent as to whether mitochondrial fission is adaptive or maladaptive. Ablation of Drp1 in mice, via the Drp1 inhibitor Mdivi-1, has a protective effect against stress induced through hypoxia and ischemia-reperfusion injury (Ong et al., 2010; Sharp et al., 2013). This could be due to the increase in mitochondrial fragmentation observed in cardiomyocytes under chronic pathological conditions (reviewed in Adaniya et al., 2019). Conversely, other studies have shown that the deletion/ablation of mitochondrial fission proteins can lead to severe cardiomyopathy (Reviewed in Hernandez-Resendiz et al., 2020).

Contrary to fission, mitochondrial fusion occurs when two separate mitochondria fuse into one, which can lead to the development of more mature mitochondrion. As such, mitochondrial fusion facilitates differentiation of cells (Kasahara et al., 2013; Lees et al., 2019). Mitochondrial fusion is facilitated by the binding of optic atrophy 1 (Opa1) protein on the inner mitochondrial membrane and mitofusin 1/2 (Mfn 1/2) in the outer mitochondrial membrane (Santel and Fuller, 2001; Chen and Chan, 2005). Studies examining the differentiation of mesenchymal stem cells (MSCs) have shown that Opa1 expression increases significantly in cells undergoing differentiation (Feng et al., 2019; Fujiwara et al., 2019) (Figure 1).

In the postnatal period, the heart shifts from relying primarily on glycolysis as a source of ATP to the oxidation of fatty acids (Lopaschuk et al., 1991). This occurs due to changes in the availability of substrates and oxygen to the heart and are supported by changes in the mitochondria (Gong et al., 2015a). Mitochondrial fusion proteins are essential in every stage of development, including both the early embryonic stage and postnatal stage (Chen et al., 2011; Papanicolaou et al., 2011; Kasahara et al., 2013). In double knockouts of Mfn1/2, there are abnormalities in mitochondrial morphology and membrane organization leading to the development of dilated cardiomyopathy as soon as 7 days post birth, and premature death in the second week of life (Papanicolaou et al., 2011). As such, it appears that Mfn1/2 is critical to the remodeling of the mitochondria postnatally in the heart. Mfn1/2 continues to be important in the adult heart, as it protects against long-term cardiac dysfunction (Chen et al., 2011).

Of importance in this context are posttranslational modifications of proteins involved in mitochondrial dynamics. Metabolism seems to have regulatory effects on both fission and fusion, with increased fusion occurring when the cell is relying on oxidative phosphorylation (Rossignol et al., 2004). The morphology of the mitochondria is also tightly linked to the metabolism of the cell. Cells that primarily utilize glycolysis for ATP production mainly have unfused spherical mitochondria (Seo et al., 2018; Zhang et al., 2018). Alternatively, fused, and matured mitochondria networks are typically present in cells that rely primarily on oxidative phosphorylation (Zhang et al., 2018; Fu et al., 2019). In cardiomyocytes, mitochondria have elongated and complex branched networks with restricted movement and are localized in three locations: 1) the intermyofibrillar spaces between contractile filaments, 2) underneath the sarcolemma, and 3) in the perinuclear region (reviewed in Adaniya et al., 2019). The importance of cristae structure on cellular functioning and metabolic shifts has been previously reviewed (Cogliati et al., 2016). Briefly, cristae are the main location of oxidative phosphorylation, encompassing 94% of complex III and ATP synthase (Gilkerson et al., 2003), and thus are critical to the energetic state of the cell. The heart has densely packed cristae, and under pathological conditions, such as hypertrophic cardiomyopathy, there is a marked decrease in cristae density and an increase in mitochondria with severely damaged cristae (Ranjbarvaziri et al., 2021). To further demonstrate the importance of mitochondrial dynamics proteins, Opa1 is essential to cristae remodeling in the event of tissue damage and the maintenance of proper function of oxidative phosphorylation *in vivo* (Civiletto et al., 2015; Varanita et al., 2015).

The mitochondria in proliferating cells, such as embryonic stem cells (ESCs) and inducible pluripotent stem cells (iPSCs), have a more immature and spherical shape (Folmes et al., 2011; Zhou et al., 2012). This is consistent with the primarily glycolytic metabolic profile that is seen in stem cells (Fillmore et al., 2015), thus indicating that there are more instances of mitochondrial fission within these cells. However, it should be noted that fusion is still important in proliferating cells, as studies have shown that deletion of Opa1 or Mfn 1/2 in neural stem cells (NSCs) leads to abnormalities in mitochondrial structure and, consequently, cellular dysfunction (Khacho et al., 2016).

Studies in which human iPSCs are differentiated to cardiomyocytes show that when Drp1 levels are blocked, thus decreasing mitochondrial fission, there is a shift toward a metabolic profile dominated by oxidative phosphorylation as opposed to glycolysis (Hoque et al., 2018). This metabolic profile, as mentioned previously, creates a more ideal environment for differentiation. Further, knockdown of Mfn2 in stem cells leads to mitochondrial respiratory dysfunction, decreases in ATP levels, and results in an overall loss in the cells’ ability to differentiate (Forni et al., 2015; Hoque et al., 2018). As such, it would appear that mitochondrial fission is decreased in the maturing cardiomyocyte, while fusion is typically seen at higher levels (Figure 1).

The regulation of energy metabolism is a key way in which cell fate is influenced by mitochondrial dynamics. While mitochondrial dynamics can also affect cell fate through various other mechanisms, such as calcium balance, this is beyond the scope of the current review (reviewed in Ren et al., 2020). Manipulation of proteins involved in mitochondrial dynamics clearly affects a cells fate and its ability to differentiate through shift in cellular metabolic profiles, indicating the importance of energy metabolism and mitochondrial dynamics on cell fate.

### Mitophagy

Mitophagy is the selective autophagy, or degradation, of mitochondria to control for quality (Lemasters, 2005), and is a very important pathway in determining cardiomyocyte fate. Mitophagy is used to remove old, damaged, and/or fragmented mitochondria, via an autophagosome, into a lysosome for degradation (Figure 1). The process of mitophagy is mediated by fission, mainly for two reasons. The first is that it is physically easier for fragmented pieces of mitochondria to be encapsulated by the autophagosome. Secondly, when fission breaks down mitochondrial networks, there is a change in the energy potential of the mitochondrial membrane. This initiates the phosphatase and tensin homolog (PTEN)-induced putative kinase 1 (PINK1)-Parkin ubiquitination pathway, which is the best-understood mechanism for mitophagy. When mitochondrial membrane potential is normal, PINK1 continuously enters the mitochondria, where it is cleaved and then translocated to the cytosol. There, PINK1 undergoes proteasomal degradation, which is why PINK1 mRNA expression is typically high, with low protein levels in normal mouse hearts (Song et al., 2015; Sekine and Youle, 2018). When the mitochondrial membrane is depolarized, as is the case during mitochondrial dysfunction, PINK1 does not undergo cleavage in the mitochondria, and collects in the OMM. This accumulation of PINK1 in damaged mitochondria both directly and indirectly attracts Parkin. PINK1 then directly phosphorylates Parkin and indirectly leads to its phosphorylation through the phosphorylation of Ubiquitin at serine 65, which leads to ubiquitination of OMM proteins (Kane et al., 2014; Koyano et al., 2014). The activation of Parkin and the subsequent ubiquitination of OMM proteins ultimately stimulate the onset the mitophagy, which is mediated through mitochondrial fission (Tanaka et al., 2010; Desai et al., 2018). Further, the ubiquitination and eventual degradation of Mfn1/2, key enzymes of mitochondrial fusion, occurs during the induction of mitophagy (Gegg et al., 2010).

In addition to the ubiquitin pathway, there are mitophagy receptors which regulate mitophagy. These receptors are characterized by microtubule-associated protein 1A/B- light chain 3 (LC3)-interacting regions (LIR). These regions directly bind LC3, which mediates autophagy by brining autophagosomes into the mitochondria. Two key receptors of mitophagy include BCL2 interacting protein 3 like (BNIP3L) and the mitochondrial receptor protein FUN14 domain-containing-1 (FUNDC1) (Figure 1). BNIP3L is essential to mitophagy as seen in the maturing erythroid cells (Sandoval et al., 2008). FUNDC1 is regulated by its state of phosphorylation (Liu et al., 2012). Dephosphorylating the serine 13 region of FUNDC1 increases the recruitment of Drp1 and degrades Opa1, and thus increases fission, furthering the association between mitophagy and mitochondrial fission (Chen et al., 2016).

Studies have shown that terminally differentiated, or adult, cardiomyocytes are not impacted by the deletion of Parkin (Song et al., 2015). This is in part due to the fact that there is already a very low presence of Parkin in the normal adult mouse heart, as seen through both mRNA and protein levels (Song et al., 2015). Interestingly, when mitochondrial fission is defective due to Drp1 ablation, it induces lethal cardiomyopathy in the mouse heart, with an upregulation of Parkin (Song et al., 2015). It is thought that the increase in Parkin leads to hyper-mitophagy and contributes the progression of cardiomyopathy by interrupting mitochondrial fission in adult hearts.

Mitophagy has an important role during the differentiating process of cardiac progenitor cells. Cardiac progenitor cells are primarily glycolytic; however, during differentiation, these cells switch to oxidative phosphorylation with concomitant increases in mitochondrial biogenesis and a more mature mitochondrial network (Chung et al., 2007; Chen C.-T. et al., 2008). Some studies indicate that mitophagy is primarily important to maintain stemness and that mitophagy is reduced during cell maturation (Ho et al., 2017; MacVicar and Lane, 2014) (Figure 1). It seems that energy metabolism of a cell plays a large part in regulating mitophagy. In RPE1 cells, changing the main energetic pathway to oxidative phosphorylation leads to a decrease in stress-induced Parkin-mediated mitophagy (MacVicar and Lane, 2014).

Others have reported an increase in mitophagy during differentiation (Zhang et al., 2009; Vessoni et al., 2012; Sin et al., 2016; Lampert et al., 2019). A potential explanation for this may be an initial increase in mitophagy during cell differentiation to remove immature mitochondria to make way for the formation of more mature mitochondrial networks (Gong et al., 2015b; Sin et al., 2016). Interestingly, it has been suggested that this is the case in the postnatal heart, in which fetal mitochondria must be removed before matured mitochondrial networks are able to form (Gong et al., 2015b). This is necessary due to the dramatic changes in metabolism from primarily glycolytic to relying on oxidative metabolism in the newborn heart (Lopaschuk et al., 1991), which requires existing mitochondria to be replaced with mitochondria that contain the necessary metabolic capacity. Lampert et al. (2019) provided evidence for the importance of mitophagy in the early stage of differentiation in cardiac progenitor cells, which was through the mitophagy receptors BNIP3L and FUNDC1, rather than the PINK-Parkin pathway (Lampert et al., 2019). In this study, mitophagy was crucial to the formation of a functional mitochondrial network (Lampert et al., 2019). When cells were unable to go through mitophagy, and rather simply went through mitochondrial fission, the mitochondria for the cell became dysfunctional, changing their morphology to a rounded donut-like shape. Zhou et al. (2020) found that glucocorticoid can induce Parkin-mediated mitophagy and promote cardiomyocyte maturation, consistent with observations by Gong et al. (2015b). As mitophagy clears out less efficient/dysfunctional mitochondria, it creates an environment for increased mitochondria biogenesis and increased mitochondrial mass consisting of mitochondria with greater quality (Zhou et al., 2020).

It would appear that mitophagy, although not high in differentiated or mature cells, is a necessary process that is a prerequisite to forming mature mitochondrial networks, as seen in differentiated/mature cells. Significantly, mitophagy seems to play a critical role in the differentiation of cells through the modulation of mitochondrial biogenesis.

### Mitochondrial Biogenesis

Mitochondrial biogenesis is a process in which mitochondria grow both in quantity and in size. An increase in mitochondrial biogenesis is critical in cell fate and the maturation of cardiomyocytes (Attardi and Schatz, 1988; Dai et al., 2017) (Figure 1). Several physiological stimuli mediate mitochondria biogenesis, including development, physical exercise, diet, and thermoregulation. Mitochondrial biogenesis is known to be critical in the control of cellular metabolism. During mitochondrial biogenesis, new mtDNA, proteins, and membranes are synthesized, and new mitochondria are formed through mitochondrial fission. Mitochondrial biogenesis is a complex process, involving proteins which stem from both mitochondrial and nuclear genomes. There are 13 mRNAs of mtDNA which encode essential proteins in the electron transport chain (ETC) and ATP synthase. However, most mitochondrial proteins (100–1,500) are encoded by nuclear genomes and synthesized in cytosolic ribosomes (Baker et al., 2007; Pagliarini et al., 2008). Peroxisome proliferator-activated receptor-γ coactivator (PGC)-1α has been identified as the main regulator of mitochondrial biogenesis, as this initiates mtDNA transcription (Untereiner et al., 2016; Wenz, 2013; Scarpulla et al., 2012). PGC1- α is activated through post-translational modification: either by phosphorylation through AMP-activated protein kinase (AMPK) or by deacetylation through sirtuin 1 (SIRT1) (Figure 1). SIRT1 increases transcriptional rates by keeping PGC-1α actively bound to chromatin (reviewed in Rodgers et al., 2008). The inhibition of SIRT1 in cardiomyocytes leads to a decrease in PGC-1α expression and an increase in reactive oxygen species (Waldman et al., 2018). Conversely, increasing SIRT1 and PGC-1α expression was found to be cardioprotective (Waldman et al., 2018). The activation of SIRT1 through nicotinamide adenine dinucleotide (NAD^+^) links mitochondrial biogenesis through the SIRT1 regulation of PGC1, with energy metabolism (reviewed in Kane and Sinclair, 2018). SIRT1 not only promotes mitochondrial biogenesis through the SIRT1/PGC-1α axis, but through the production of nitric oxide synthase (NOS) via endothelial NOS activation (Csiszar et al., 2009). The activation of PGC-1α through phosphorylation via AMPK further provides a link between energy metabolism and mitochondrial biogenesis (Jager et al., 2007). PGC-1α activation stimulates nuclear transcription factors: nuclear respiratory factor (NRF)-1, NRF-2, and estrogen related receptor α (ERR- α). NRF-1 activates mitochondrial transcription factor A (TFAM) leading to increased mtDNA transcription and replication (Gleyzer et al., 2005) (Figure 1). This leads to an increase in mitochondrial proteins, and mitochondrial biogenesis. NRF-1 and NRF-2 regulate the expression of many complexes in the ETC (Scarpulla, 2008; Satoh et al., 2013). When there is a genetic loss of NRF-1 or NRF-2, it results in embryonic fatality, with a reduction in mtDNA ETC activity (Huo and Scarpulla, 2001; Ristevski et al., 2004). ERRα regulates transcription of the gene encoding for medium-chain acyl-CoA dehydrogenase (MCAD), which is an enzyme that mediates the initial steps of mitochondrial fatty acid oxidation (FAO) (Sladek et al., 1997; Vega and Kelly, 1997). ERRα works similarly to PGC-1α, in that it is involved in every pathway of mitochondrial energy transduction and ATP synthesis, such as fatty acid oxidation, the tricarboxylic acid (TCA) cycle, and oxidative phosphorylation (Huss et al., 2004). ERRγ has also been identified as an important transcription factor in the maturation of cells, as cardiac-specific deletions of ERR α/ERRγ lead to reduced expression of genes necessary for mitochondrial energy production (Wang et al., 2015). Additionally, it would appear that Parkin, a key regulator of mitophagy, also plays an important role in the regulation of mitochondrial biogenesis (Kuroda et al., 2006; Rothfuss et al., 2009; Vincow et al., 2013). In *Drosophila* with Parkin deficiency, mtDNA content decreases in *Drosophila* heart tubules with an overall reduction in mitochondrial biogenesis (Bhandari et al., 2014).

## The Role of Reactive Oxygen Species in Cardiomyocyte Differentiation

During the differentiation of cardiomyocytes during postnatal development, there is an observed increase in mitochondrial oxidative metabolism (Lopaschuk et al., 1991). This shift toward oxidative metabolism is accompanied by increased intracellular reactive oxygen species (ROS) (Hom et al., 2011), a by-product of mitochondrial metabolism, which appears to play a significant role in cardiomyocyte differentiation. Reactive oxygen species is primarily generated through the ETC and the nicotinamide adenine dinucleotide phosphate (NADP) oxidase (NOX) complex (Qu et al., 2016). Typically, stem cells exist in environments with low ROS production, which maintains their stemness and ability to self-renew (reviewed in Wei and Cong, 2018). When ROS is increased, there is a reduction in self-renewal and a greater potential for differentiation (Pervaiz et al., 2009). ESC differentiation is impaired when the ETC is inhibited, and subsequently when ROS is reduced (Wei and Cong, 2018). Studies have demonstrated that in particular, NOX-dependent ROS is critical for the differentiation of ESCs into cardiomyocytes, as it induces the expression of cardiac genes (Murray et al., 2013). ROS regulates gene expression through chromatin and histone modifications, which is essential for cardiac development (Wei and Cong, 2018). The NOX isoform NOX_4_ is the primary isoform involved in cardiomyocyte differentiation (Wei and Cong, 2018). In the earliest stages of cardiomyocyte differentiation, NOX_4_ stimulates the cardiac transcription of *GATA-4* through the c-Jun transcription factor (Murray et al., 2013). Additionally, an ESCs ability to differentiate and express cardiac markers is reduced when NOX_4_ is downregulated through siRNA knockdown (Ding et al., 2008). Interestingly, it appears that there is a certain balance of acceptable ROS at particular stages of development that is necessary to promote differentiation, as too much or too little can have inverse affects (Wei and Cong 2018). ROS is higher in the earlier stages of cardiomyocyte differentiation; however, application of antioxidants tended to promote further differentiation (Hom et al., 2011).

## Mitochondrial Biogenesis and Maturation on Cardiomyocyte Cell Fate

Mitochondria mature through a combination of mitochondrial fission, fusion, mitophagy, and mitochondrial biogenesis. This section will discuss the role that mitochondrial biogenesis has on the differentiation and maturation of cardiomyocytes. This will be discussed in the context of the developing postnatal heart, as well as in the context of stem cells differentiating into cardiomyocytes.

### Postnatal Cardiomyocyte Maturation

Cardiac energy demands are dynamic during developmental stages; as a result, mitochondrial number and function must change to support these changes in energy demand (Attardi and Schatz, 1988). During the early postnatal period, myocardial energy demand increases dramatically; as such, there is an observed increase in the number of mitochondria in the cell and hence an increase in mitochondrial proteins (Mayor and Cuezva, 1985; Attardi and Schatz, 1988). Cardiac mitochondria are characterized by their ability to efficiently produce ATP through oxidative metabolism (reviewed in Stanley et al., 2005). As previously discussed, increases in mitochondrial number and proteins are associated with increased mitochondrial biogenesis; this is mainly regulated through PGC-1 coactivators, such as PGC-1α and PGC-1β, which seem to have over-lapping roles in the heart in the immediate postnatal stage (Lai et al., 2008). In mouse hearts, PGC-1 gene expression is increased directly after birth; this is observed before increases in mitochondrial biogenesis, or the switch of energy production from relying mainly on glucose to relying on fatty acids (Lehman et al., 2000). If PGC-1 is overexpressed in primary rat neonatal ventricular cardiomyocytes, there is an increase in mitochondrial biogenesis and oxidative phosphorylation. If PGC-1 is overexpressed *in vivo* in transgenic mice, there is an uncontrollable mitochondrial biogenesis, with the mice presenting with massive edema, increased heart size, and cardiomyopathy (Lehman et al., 2000). Therefore, it seems that there is a certain threshold of PGC-1 expression needed to properly regulate the heart’s development.

When PGC-1α or PGC-1β is knocked out in mice, it was observed that, while the animals lived, there was a decrease in the growth of the heart and slow-twitch skeletal muscle, both of which have higher energy demand (Leone et al., 2005; Lelliott et al., 2006). If a PGC1α/β double knock out is produced, the mice die soon after birth, and they were observed to have smaller hearts, irregular rhythms, and reduced cardiac output (Lai et al., 2008). Martin et al. (2014) created a viable PGC1-α/β-deficient mouse through the deletion of PGC-1β in the heart and skeletal muscle through the actions of Cre recombinase regulated by the muscle creatine kinase (MCK) promoter (MCK-Cre) with a generalized PGC-1α null background (PGC-1α^−/−βf/f/MCK−Cre^). They showed that the loss of both PGC-1α and PGC-1β during postnatal development results in lethal cardiomyopathy due to defects in mitochondrial maturation and reduced expression of genes associated with mitochondrial dynamics (Martin et al., 2014). Interestingly, in this PGC-1α^−/−βf/f/MCK−Cre^ mouse, mitochondrial biogenesis still increased 1 day post birth; however, significant morphological abnormalities were seen by the first week, with mitochondria becoming donut-shaped by 8 weeks of age, with a reduction in mitochondrial density and networks (Martin et al., 2014). Additionally, these changes were associated with a decrease in mtDNA, ETC complexes I and IV. Consistent with other studies, when only a single PGC-1 was deleted, there were no significant abnormalities in structure and function. As expected with these changes in morphology, Martin et al. (2014) also observed decreased *Mfn1 and Opa1*, key genes associated with mitochondrial fusion (Scarpulla et al., 2012; Kasahara et al., 2013). As previously discussed, mitochondrial dynamics and mitophagy play a key role in mitochondrial maturation and biogenesis, and this is further confirmed by this PGC-1α/β deletion study. The reduction in *Mfn1* seen in the PGC-1 deletion is most likely due to an associated decrease in ERRα, as ERRα promotes *Mfn1* transcription (Martin et al., 2014). Both ERRα and ERRγ are important in postnatal cardiac development, as it directly activates metabolic and structural genes, all while suppressing fetal and non-cardiac myocyte genes (Sakamoto et al., 2020). Further, Martin et al. (2014) demonstrated that the deletion of PGC-1α/β did not affect mitochondrial dynamics in adult mouse hearts. This demonstrates the importance of PGC-1α and PGC-1β in the newborn period, as this is where the most dramatic effects on mitochondrial maturation and biogenesis are seen. Together, studying the overexpression and deletion of PGC-1s lends to the importance of PGC-1 coactivators, particularly PGC-1α and PGC-1β, in the maturation of cardiomyocytes post birth. A physiological expression of PGC-1s must be maintained for the heart to develop and function normally.

### Stem Cells

Stem cells are non-specialized cells, which, under certain conditions, can mature into cells with a specific function. Given the context of this review, this section will discuss the role that mitochondrial maturation plays in the ability of stem cells to differentiate into cardiomyocytes. In the mature cardiomyocyte, mitochondria take up a significant amount of cell space, occupying 20–40% of cell volume (Schaper et al., 1985). During prolonged culture of human iPSC-derived cardiomyocytes (hiPSC-CMs), there is an increase in mitochondrial biogenesis, as well as increases in membrane potential ETC complex activity (Dai et al., 2017). In line with this increase in mitochondrial biogenesis, within hiPSCs-CMs, there is a significant upregulation of PGC-1α, which promotes a more mature metabolism consistent with cardiomyocytes (Venkatesh et al., 2021). ZLN005, a chemical which increases PGC-1α gene expression, will promote the maturation of cardiomyocytes derived from human embryonic stems cells (hESCs) (Liu et al., 2020). This occurs due to an upregulation of TFAM and NFR1 gene expression and ERRγ mRNA; additionally, there is a 1.5-fold increase in mtDNA copy number as well as an increase in mitochondrial mass (Liu et al., 2020). This indicates the importance of PGC-1α activation in a stem cell’s ability to differentiate into a cardiomyocyte.

Mitochondrial dynamics also plays a key role in the development of cardiomyocytes from stem cells. The morphology of the mitochondria changes dramatically from granular shapes in iPSCs and ESCs, to tubular networks in differentiated cardiomyocytes (Kasahara et al., 2013; Hoque et al., 2018). Key regulators of mitochondrial fusion are increased during the differentiation of ESCs to cardiomyocytes, with a decrease in the mRNA of mitochondrial fission proteins, such as Drp1 (Kasahara et al., 2013; Hoque et al., 2018). In support of this, if Drp1 is decreased through gene trapping, there is an impairment in the ability of ESCs to differentiate to cardiomyocytes; this seemed to be arbitrated by stimulation of calcineurin-A mediated Notch signaling (Kasahara et al., 2013). As such, activation of calcineurin-A is able to block ESC differentiation into cardiomyocytes. Additionally, mitochondrial fusion can be stimulated to differentiate iPSCs through the inhibition or gene silencing of the mitochondrial fission regulator, Drp1 (Hoque et al., 2018).

Together, it is clear that mitochondrial biogenesis and mitochondrial dynamics have an important role in the maturation of mitochondria (Figure 1). Clearly, the regulation of these pathways is important to the maturation of and/or differentiation to cardiomyocytes.

## How Energy Metabolism Affects Cell Fate With a Focus On the Implication for Cardiomyocyte Fate

Mitochondria regulate cellular metabolism to match the energy needs of the cell. However, energy metabolism also plays a role in regulating the fate of a cell. In the following section, the roles of glycolysis, glucose oxidation, fatty acid oxidation, ketone oxidation and glutamine oxidation in the regulation of cell maturation will be discussed with a focus on their influence in cardiomyocyte maturation.

### Glucose Metabolism

#### Glycolysis

**FIGURE 2:**
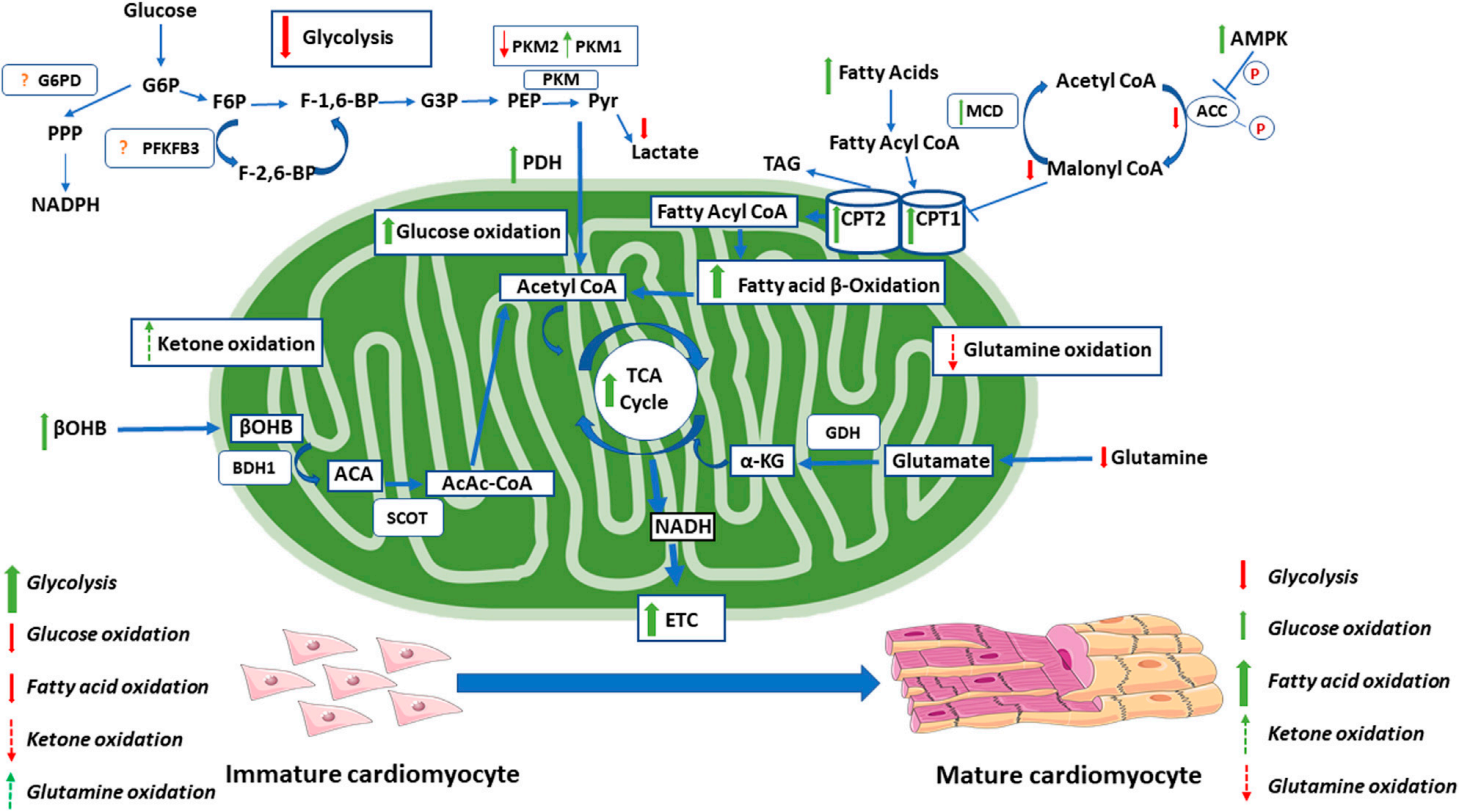
Changes in energy metabolism during the maturation of the cardiomyocyte. During the maturation of the cardiomyocyte, there are significant changes in energy metabolism. This includes increase in oxidative metabolism, with moderate increases in glucose oxidation and dramatic increases in fatty acid oxidation. During this switch, there is also a decrease in glycolytic rates. Red arrows indicate decreases seen with maturation. Green arrows indicate increases seen with maturation. Dashed red arrows indicate proposed decreases seen with maturation (not definitely known). Dashed green arrows indicate proposed increases seen with maturation (not definitely known). The exact changes in the glycolytic regulation through PFKFB3 and G6PD within the maturing cardiomyocyte is not clear and requires further investigation. Acetoacetate (ACA). Acetoacetyl CoA (AcAc-CoA). Acetyl-CoA carboxylase (ACC). AMP- activated protein kinase (AMPK). Beta-hydroxybutyrate dehydrogenase 1(BDH1). Beta-hydroxybutyrate (βOHB). Carnitine palmitoyl transferase (CPT1). Electron transport chain (ETC). Fructose-1,6- bisphosphate (F-1,6-BP). Fructose-2,6-bisphosphate (F-2,6-BP). Fructose-6-phosphate (F6P). Glucose-3-phosphate (G3P). Glucose-6-phosphate (G6P). Glucose-6-phosphate dehydrogenase (G6PD). Glutamate dehydrogenase (GDH). Malonyl-CoA decarboxylase (MCD). Nicotinamide dinucleotide phosphate hydrogen (NADPH). Nicotinamide dinucleotide hydrogen (NADH). Pyruvate dehydrogenase (PDH). Phosphoenolpyruvate (PEP). 6-phosphofructo-2-kinase/Fructose-2,6-bisphosphate (PFKFB3). Pyruvate kinase isoform M1/2 (PKM 1/2). Pentose phosphate pathway (PPP). Pyruvate (Pyr). Succinyl-CoA:3-oxo-acid CoA-transferase (SCOT). Tricarboxylic acid (TCA). Triacyl glycerol (TAG).

High rates of glycolysis are a common characteristic of proliferating cells, including fetal cardiomyocytes, stem cells, and tumor cells. The high glycolytic rates are an alternative source of energy (ATP) production for proliferating cells that have an immature mitochondrial morphology, decreased mitochondrial respiration and lower oxidative reserve capacity compared to differentiated cells (Folmes et al., 2011; Cho et al., 2006; Kondoh et al., 2007, Folmes et al., 2012a). During differentiation of cells, such as maturation of cardiomyocytes following birth, there is a dramatic decrease in glycolysis as mitochondrial oxidative metabolism increases (Lopaschuk et al., 1991) (Figure 2). In contrast, increases in glycolytic enzymes are observed during nuclear reprogramming toward a more pluripotent or stem-like state (Folmes et al., 2011; Hansson et al., 2012). In long-term hematopoietic stem cells (LT-HSCs), there are high levels of glycolytic intermediates (i.e., fructose-1,6-bisphosphate, and pyruvate), which are the products of the rate-limiting steps of glycolysis (Simsek et al., 2010). LT-HSCs also have a high expression of pyruvate dehydrogenase kinase (PDK) 2 and 4, enzymes which phosphorylate and inactivate pyruvate dehydrogenase (PDH), essentially uncoupling glycolysis from glucose oxidation (Takubo et al., 2013). This is seen through functional exhaustion when PDK 2 and 4 are deleted from HSCs, and the re-initiation of the cell cycle when glycolysis is subsequently restored (Takubo et al., 2013). Although glycolysis is a much less efficient way to generate ATP compared to glucose oxidation, it does allow for ATP to be generated quickly and in the absence of oxygen (Pfeiffer et al., 2001). Proliferating cells are not limited by ATP production as glycolytic cells are able to maintain a high ATP/ADP ratio even during division (DeBerardinis et al., 2008; Christofk et al., 2008b). However, high rates of glycolysis are advantageous to rapidly dividing cells, as it allows for the maintenance of intermediates required for the biosynthesis of cellular content for the daughter cells (Vander Heiden et al., 2009; Lunt and Vander Heiden, 2011). Pentose phosphate pathway (PPP) intermediates, such as ribose-5-phosphate, provide the carbons for purine and pyrimidine nucleotides, amino acid, and triacylglycerol/lipid synthesis (Lunt and Vander Heiden, 2011). The premature inhibition of glycolysis in stem cells can lead to cell death, whereby stimulating glycolysis through the inhibition of oxidative phosphorylation maintains the stemness of a cell and reduces its ability to differentiate (Kondoh et al., 2007; Varum et al., 2009). During the differentiation of cells from embryonic cells, such as in the case of the developing retina, a switch from glycolysis to oxidative phosphorylation is required for proper development (Agathocleous et al., 2012). To support the ATP needs of differentiated cells, there is an upregulation in TCA cycle enzymes ETC subunits (Chung et al., 2007; Tormos et al., 2011).

#### Glucose Oxidation

Differentiated cells, such as cardiomyocytes, require more ATP to sustain specialized functions such as contractions (Folmes et al., 2012b). As such, there is a transition from having high rates of glycolysis, to increased mitochondrial oxidative phosphorylation (Figure 2). This is supported by increases in TCA cycle ETC enzymes/subunits which allows for greater ATP production (Chung et al., 2007; Tormos et al., 2011). This shift is necessary for differentiation, as blocking the ETC leads to an impaired ability of ESCs to differentiate (Mandal et al., 2011). Further, when key enzymes of glycolysis and the PPP are inhibited, myogenic differentiation is stimulated (Bracha et al., 2010).

The switch from glycolysis to mitochondrial oxidative phosphorylation as a source of ATP production in the maturing cell would be expected to be accompanied by an increase in glucose oxidation. Curiously, however, during the initial maturation of cardiomyocytes, there is no substantial increase in glucose oxidation, but rather an increased oxidation of other energy substrates such as fatty acids (Lopaschuk et al., 1991). Glucose oxidation in the newborn heart does not typically increase until weaning (Lopaschuk et al., 1991). What is responsible for this delayed maturation of glucose oxidation is not clear, although competition between glucose oxidation and fatty acid oxidation as a source of TCA acetyl CoA may contribute to these low rates of glucose oxidation.

An important step in glucose oxidation is the initial mitochondrial uptake of pyruvate derived from glycolysis by the mitochondrial pyruvate carrier (MPC) complex, which transfers pyruvate into the mitochondria to undergo further metabolism. MPC is found in low levels in intestinal stem cells, and in high amounts when these cells are differentiated (Schell et al., 2017). When glucose oxidation is blocked through the of deletion of MPC, there is an increase in stem cell number and proliferation (Schell et al., 2017). Similarly, if PDH, a key enzyme that converts pyruvate to acetyl CoA, is disrupted with RNAi, there is an increase in ISC proliferation (Schell et al., 2017). This indicates the important role that glucose oxidation plays in the regulation of stem cell fate, as inhibiting glucose oxidation through the inhibition of MPC or PDH leads to an increased stem-like state with increased proliferation.

Once stem cells differentiate, they become metabolically more active, and depend more on oxidative phosphorylation for energy production compared to glycolysis (Zhang et al., 2018; Fu et al., 2019). As previously mentioned, these metabolic shifts are tightly linked to mitochondrial dynamics. If Drp1 is blocked in hiPSCs differentiating into cardiomyocytes, there is an inhibition of mitochondrial fission and a shift from glycolysis toward oxidative phosphorylation (Hoque et al., 2018).

#### The Warburg Effect

The Warburg effect is a metabolic state in which there are high rates of glycolysis uncoupled from glucose oxidation under aerobic conditions, leading to increased production of lactate. The Warburg effect is typically seen in cancerous cells and actively proliferating cells (Abdel-Haleem et al., 2017), but is also present in fetal proliferating cardiomyocytes (Lopaschuk et al., 1991) and hypertrophied myocardium (see Lopaschuk et al., 2010 for review). In cancer cells, a high Warburg effect promotes their growth, survival, and proliferation. Given that stem cells have a high rate of glycolysis, even in the presence of oxygen, they also exhibit the Warburg effect (Chung et al., 2007; Folmes et al., 2011; Kondoh et al., 2007). This is thought to be due to a low copy number of mtDNA within stem cells, leading to more immature mitochondria (Chen C. et al., 2008; Folmes et al., 2011). As previously mentioned, although the Warburg effect is inefficient in its ability to produce ATP, the carbons from glucose can be used for anabolic processes needed to support cell proliferation (DeBerardinis et al., 2008; Vander Heiden et al., 2009; Boroughs and DeBerardinis, 2015). Particularly, there is a greater synthesis of reducing equivalent in the PPP, such as reduced NADPH, which is highly consumed during the synthesis of amino acids and nucleotides needed to replicate cellular content during division (Lehninger et al., 1993; Vander Heiden et al., 2009). Additionally, a characteristic of the Warburg effect is that the fate of pyruvate to lactate production is higher, leading to the regeneration of NAD^+^ from NADH, which further supports proliferation and allows for the continuation of glycolysis (Vander Heiden et al., 2009). When stem cells differentiate and mature, there is a reversal of the Warburg effect, as evidenced by a decrease in glycolysis and an increase in oxidative phosphorylation, as discussed above, to better support the needs of the differentiated cells (Shyh-Chang et al., 2013).

The fetal heart’s metabolism reflects the Warburg effect, as there is a high dependency on glycolysis for energy production. This is mediated through hypoxia-inducible factor 1-alpha (HIF-1α) that is generated due to the fetal heart’s location within a low-oxygen environment (Lopaschuk et al., 1992; Makinde et al., 1998). As soon as 7 days post-birth, there is a decrease in glycolysis such that it contributes to only 10% of ATP production (Lopaschuk et al., 1991; Medina, 1985). This reduction in glycolysis is indicative of a reduced Warburg effect. Interestingly, glucose oxidation still remains low during this time, most likely due to an increase in PDK expression, which phosphorylates and inhibits PDH (Lopaschuk et al., 1991). As discussed previously, glucose oxidation does not fully mature in the heart until the infant is weaned (Girard et al., 1992). Rather, the heart switches from primarily glycolytic metabolism to fatty acid oxidation immediately post birth (Lopashuk et al., 1991). This switch is also seen in hiPSC-CMs (Nose et al., 2018). Exposure to high levels of glucose leads to an impaired differentiation into cardiomyocytes, seen in both human and mouse ESCs (Yang et al., 2016; Nakano et al., 2017). Conversely, suppressing glucose levels supplements the differentiation and maturation of hiPSC-CMs (Nakano et al., 2017). Together, this indicates the reliance that proliferating stem cells or immature cardiomyocytes have on the Warburg effect, and that this is reversed during differentiation and maturation.

Pyruvate Kinase isoform M2 (PKM2), 6-phosphofructo-2-kinase/Fructose-2,6-bisphosphate (PFKFB3), and glucose-6-phosphate dehydrogenase (G6PD) have been identified as key regulators of the Warburg effect in proliferating cells (Figure 2). Pyruvate kinase dephosphorylates phosphoenolpyruvate during glycolysis, producing ATP and pyruvate. As discussed previously, anabolic metabolism is integral to proliferative capacity. PKM2 is mainly expressed in proliferating cells; however, it has lower enzymatic activity compared to PKM1 which is mainly expressed in adult cells (Garnett et al., 1974; Christofk et al., 2008a). In proliferating cells, PKM2 exists in a dimer conformation, which has a lower enzymatic activity and promotes an increase in anabolic metabolism, through the PPP, which subsequently allows for the synthesis of biomolecules necessary for proliferation (Ikeda and Noguchi, 1998). PKM2 can shift into an active tetrameric conformation by upstream F-1,6-BP (Ashizawa et al., 1991). In contrast, phosphotyrosine-marked proteins revert PKM2 to a lower activity conformation, through a dissociation of F-1,6-BP (Christofk et al., 2008b). As such, small molecule activators of PKM2 have been studied as a way to induce the active tetrameric conformation of PKM2, which resemble the effects of PKM1 substitution studies, leading to decreased tumorgenicity (Christofk et al., 2008a; Kung et al., 2012). Overall, studies in which PKM2 is activated to its tetrameric conformation were shown to lead to a decrease in the cells ability to proliferate (Anastasiou et al., 2012; Kung et al., 2012; Parnell et al., 2013). PKM2 also seems to play a role in the fate of pyruvate, as it can be reduced to lactate or oxidized to acetyl-CoA for further pyruvate oxidation. Increased PKM2 activity leads to an increase in the amount of pyruvate being used for mitochondrial oxidative metabolism, and a reduced production of lactate (Christofk et al., 2008a). This may be due to an increase in PKM2 binding to Mfn2, which promotes mitochondrial fusion, through mTOR, which subsequently leads to an increase in oxidative phosphorylation and a decrease in glycolysis (Li et al., 2019). While the knockdown of PKM2 decreases glycolytic activity in cancer cells, increasing PKM2 is able to restore both glycolysis and oxidative phosphorylation (Li et al., 2019). Notably, in glycolysis defective mutated PKM2, restoration of PKM2 in PKM2 deficient cells still leads to a partial increase in oxidative phosphorylation, indicating that PKM2s regulation of oxidative metabolism is partially independent of its effects on glycolysis (Li et al., 2019). During cardiomyocyte development, PKM2 appears to have a significant role, as it is highly expressed during development and immediately after birth, although it is replaced by PKM1 in the adult cardiomyocyte (Magadum et al., 2020). In PKM2 deletion studies, myocardial size and cardiomyocyte quantity are reduced (Magadum et al., 2020). The issue of whether enhanced or lowered PKM2 is important in inducing proliferation post myocardial infarction (MI) has produced controversial results. Magadum et al. (2020) suggest that an increase in PKM2 is related to an increase in proliferation and cardiomyocyte regeneration, whereas Hauck et al. (2020) suggest the opposite, in which a reduction in PKM2 expression would be more beneficial for cardiomyocyte regeneration post MI. Notably, neither of these studies demonstrates whether their results were aligned with the dimer or tetramer conformation of PKM2. As such, more research regarding the role of PKM2 in cardiomyocyte proliferation and differentiation is necessary.

The role of PFKF3B in cell fate has been mainly studied in the context of cancer and angiogenesis (the process of forming new blood vessels). PFKFB3 is responsible for an increased synthesis of fructose-2,6-bisphosphatase (F-2,6-BP), which allosterically regulates phosphofructkinase-1 (PFK-1) (Pilkis et al., 1981; Uyeda et al., 1981; Pilkis et al., 1995). PFK-1 catalyzes a key rate-limiting step of glycolysis, the conversion of fructose-6-phosphate to F-1,6-BP (Ros and Shulze, 2013). PFKFB3 is associated with an increase in glycolysis within cancer cells, as seen by increases in PFKFB3 expression and phosphorylation (Novellasdemunt et al., 2012). It has been shown that F-2,6-BP is upregulated in cancerous cells, associated with stimulation of glycolysis, and its depletions can suppress cell survival and proliferation (Hue and Rousseau. 1993; Nissler et al., 1995). As such, the inhibition of PFKFB3, and the subsequent decrease in F-2,6-BP, has been examined to determine its effects on tumor growth and cell death (Seo et al., 2011). PFKFB3 inhibition/silencing indeed leads to a decrease in F-2,6-BP, subsequently reducing glycolysis and resulting in an inhibition of tumor growth and an induction of cancer cell death (Chesney et al., 1999; Seo et al., 2011; Clem et al., 2008). *Cancer* stem cells (CSCs) are a type of cells within tumors that are capable of self-renewal and differentiation (Wicha et al., 2006), and can develop new tumors (Rhim et al., 2012). These CSCs are typically resistant to cancer treatments (Dean et al., 2005; Vaz et al., 2014). High levels of PFKFB3 are characteristic of CSCs compared to iPSCs and other cancer cells (Cieślar-Pobuda et al., 2015). This is important because in the process of transplanting iPSCs, there is the possibility of cancerous tumor formation due to the presence of undifferentiated IPSCs (Knoepfler, 2009). As such, being able to differentiate between CSCs and normal stem cells is critical to the use of iPSCs in regenerative medicine. PFKFB3 has also been studied in endothelial cells (ECs) during angiogenesis. ECs consume high levels of glucose and exhibit the Warburg effect, as they are highly glycolytic, even in the presence of oxygen (Dobrina and Rossi, 1983; De Bock et al., 2013). This aligns with the proliferative nature of ECs. When PFKFB3 is silenced in ECs, there is a reduction in vessel sprouting/proliferation, which affect both the migrating tip and the proliferating stalk of ECs (De Bock et al., 2013). This is due to a decrease in the ability of the ECs to metabolize glucose through glycolysis, indicating again the importance of PFKB3 in the regulation of glycolysis in proliferating cells. Together, these bodies of evidence make PFKFB3 an interesting target of the Warburg effect, and further research should be done in understanding its role in the maturing and differentiating cardiomyocyte.

As previously described, high levels of anabolic metabolism and the biosynthesis of cellular content are required for rapidly dividing and proliferating cells. This PPP is critical in this process. G6PD temporarily shunts glucose from the glycolytic pathway to the PPP, where it leads to the production of ribose and NADPH. These products are critical for biosynthesis and anabolic metabolism. As described by Yang et al. (2019), several studies have shown an increase in G6PD activity in various different cancer cell lines. Knockdown of G6PD decreases cancer cell proliferation and glycolysis, while reducing the tumorigenic properties of gastric cancer cells (Deng et al., 2021). In non-cancerous cells, reduced G6PD activity blocks regular proliferation and can lead to deficiencies in growth and development in animal models (Tian et al., 1998; Li et al., 2009; Yang et al., 2013). The mammalian knockout of G6PD leads to embryonic lethality (Longo, 2002). Increasing PPP activity has been associated with increased and more aggressive tumor malignancy (Richardson et al., 2008). G6PD is regulated by the NADPH/NADP ratio, such that a reduction in NADPH activates G6PD (Kletzien et al., 1994). G6PD is also regulated through transcription, translation, post-translational modification, as well as numerous positive and negative regulators (Stanton, 2012). However, a detailed description of these regulations is beyond the scope of this review. Importantly, along with leading to the production of important components for proliferation, NADPH is essential in protecting cells from oxidative stress as described by Yang et al. (2019). Briefly, NADPH is a potent antioxidant, and the knockout of G6PD leaves embryonic stem cells highly sensitized to oxidants, such as diamide, ultimately resulting in increased cell death (Pandolfi et al., 1995). NADPH is essential to the proper functioning of the major components of the antioxidant system, the glutathione system, catalase, and superoxide dismutase, either through NAPDHs reductive properties, or through allosteric binding (Zhang et al., 2010). In the cardiomyocyte, the pentose/G6PD/NADPH/glutathione pathway seems to have a cardioprotective mechanism, as it protects against ROS-induced injury, and deficiencies in G6PD lead to increased MI-induced damage in animal models (Jain et al., 2004; Wu et al., 2004). Indeed, in hypertrophied cardiomyocytes, there is a decrease in G6PD expression (Li et al., 2020). Moreover, restoring G6PD activity prevents the dysregulation of mitochondrial function and oxidative stress experienced by these cells (Li et al., 2020). This indicates the importance of G6PD in adult cardiomyocytes. Of note, this seems to be at odds with the high activity of G6PD also seen in proliferative cancer cells. Considering that differentiated cells seem to have almost opposite metabolic profiles compared to proliferating cells, further research needs to be done looking at the role of G6PD in cardiomyocyte development and differentiation.

### Fatty Acid Oxidation

Mitochondrial fatty acid oxidation plays an important role in cardiomyocyte maturation. A dramatic increase in fatty acid oxidation occurs in the maturing heart following birth (Lopaschuk et al., 1991). Interestingly, a decrease in fatty acid oxidation is seen in the stressed heart, such as that seen with congenital heart defects, which maintains a more fetal metabolic and contractile phenotype (Lopaschuk et al., 2010; Lam et al., 2015). In the transition from the fetal to newborn period, there are significant changes in energy metabolism (Lopaschuk et al., 1991). Fatty acid oxidation is low in the fetal heart as a result of the low levels of fatty acids present (Girard et al., 1992). In the newborn period, there is a shift in the metabolic profile to sustain the cellular growth that occurs during this period (Soonpaa et al., 1996; Ahuja et al., 2007). This includes a shift toward increased fatty acid oxidation, which produces the majority of ATP in the newborn and supports the increased requirement for ATP from the rapidly growing and beating heart (Lopaschuk et al., 1991; Itoi and Lopaschuk, 1993) (Figure 2). This is similar to the adult heart in which the majority of ATP produced is obtained from mitochondrial oxidative phosphorylation, with 60–90% of acetyl-CoA being produced from fatty acid oxidation and 10–40% being derived from the oxidation of pyruvate (Stanley et al., 2005; Kolwicz et al., 2013). Of the fatty acids that enter adult cardiomyocytes, 70–90% are converted to acylcarnitine for oxidation via carnitine palmitoyl transferase 1 (CPT-1), and the other 10–30% enter the intracardiac triacylglycerol pool (Saddik and Lopaschuk, 1991). Fatty acid oxidation is transcriptionally regulated by the PGC-1α/PPARα pathway (Huss and Kelly, 2004; Finck, 2007). PPARα forms heterodimers with retinoid X receptor (RXR), which regulates the expression of genes involved in fatty acid activation (van der Lee et al., 2000), uptake (Brandt et al., 1998; Mascaró et al., 1998; van der Lee et al., 2000), and oxidation (Gulick et al., 1994; Leone et al., 1999; van der Lee et al., 2000). PGC-1α also transcriptionally coactivates PPARβ/δ, although there is a high degree of redundancy with PPARα in terms of its regulation of fatty acid oxidation (Gilde et al., 2003). This is consistent with increased levels of PGC-1α, and PPARα/β expression in the postnatal heart (Panadero et al., 2000; Lai et al., 2008; Martin et al., 2014).

Mitochondrial fatty acid uptake is necessary for fatty acid oxidation, and this uptake is mediated by CPT-1 (Lopaschuk et al., 1994a). CPT1 is regulated through the inhibitory effects of malonyl-CoA, which is a key regulator of cardiac fatty acid oxidation (Lopaschuk et al., 1994a; McMillin et al., 1994). Malonyl-CoA levels are also a key regulator of fatty acid oxidation in the newborn period, with levels decreasing rapidly in the days after birth (Lopaschuk et al., 1994b; Lopaschuk and Gamble, 1994). Reduced levels of malonyl-CoA occur due to both a decrease in synthesis and an increase in degradation (Lopaschuk et al., 1994b; Dyck et al., 1998). Malonyl-CoA synthesis is catalyzed by acetyl-CoA carboxylase (ACC), an enzyme which is phosphorylated and subsequently inactivated after birth by AMPK (Hardie, 1989; Hardie, 1992; Saddik et al., 1993; Lopaschuk et al., 1994b). AMPK also acts as an activator of PGC-1α, which also leads to an increase in fatty acid oxidation (Jager et al., 2007). Malonyl-CoA is also reduced due to its degradation through decarboxylation by Malonyl-CoA decarboxylase (MCD) (Dyck et al., 1998; Dyck et al., 2000). MCD expression is high in the neonatal heart which, along with the inhibition of ACC, leads to a decrease in malonyl-CoA and an increase in fatty acid oxidation (Sakamoto et al., 2000; Yatscoff et al., 2008).

Evidence suggests that hiPSC-CMs do not display adult cardiomyocyte metabolism, but rather maintain more fetal cardiomyocyte characteristics (Mummery et al., 2012; Robertson et al., 2013; Yang et al., 2014). However, incubating hiPSC-CMS with fatty acid increases maturation, as seen through improvements in morphology, protein expression, and metabolism (particularly through an increase in fatty acid oxidation) (Drawnel et al., 2014; Horikoshi et al., 2019). During the maturation of hiPSC-CMs, PGC-1α is a major upstream regulator, which is a known transcriptional regulator of fatty acid oxidation (Venkatesh et al., 2021). Additionally, treatment of hESC-derived cardiomyocyte (hESC-CMs) with retinoic acid upregulates fatty acid oxidation, through the activation of retinoic acid receptor/RXR and PPAR/RXR heterodimers (Miao et al., 2020). This increase in fatty acid oxidation was further evidenced by an overexpression of PDK4, which inhibits PDH activity, and an increased expression of PGC-1α, which leads to an increase in ATP production through fatty acid oxidation (Miao et al., 2020).

### Ketone Oxidation

The role of ketone oxidation in the regulation of cell fate has not been extensively studied. However, emerging evidence suggest a key role for ketones in the regulation of cell fate, such as in cancer (Singh et al., 2015; Ferrere et al., 2021). The increased presence of ketone bodies in the immediate postnatal period also lends to the potential role it may have in regulating cardiomyocyte fate. Ketogenic diets (KD) use a high-fat and low-carbohydrate diet to increase levels of circulating ketone bodies, the main ketone in humans being ß-hydroxybutyrate (βOHB). βOHB has been shown to have an anti-tumor affect in tumor models, through the regulation of the immune system (Ferrere et al., 2021). The KD has also been shown to decrease tumor proliferation by decreasing rates of glycolysis (Singh et al., 2015). A potential mechanism for this is through the inactivation of the insulin/IGF-1-dependent phosphatidylinositol 3-kinase (PI3K)/Akt/mTOR pathway and the activation of AMPK (Weber et al., 2018). It should also be noted that the KD leads to increased mitochondrial enzymes and protein content, as well as increased fatty acid oxidation (Sparks et al., 2005; Jensen et al., 2011; Li et al., 2016). Studies on neurodegenerative disorders have found that treatment with KD leads to a PGC-1α regulated increase in mitochondrial biogenesis (Hasan-Olive et al., 2019). This provides evidence that the KD can manipulate the mitochondria, the regulation of which plays a large role in determining cell fate. Additionally, in the immediate newborn period, there is an increase in circulating ketones which provides an additional metabolic substrate during this time of cellular development. (Bougneres et al., 1986).

βOHB is not only a fuel source for the heart but also has cell signaling properties. One signaling pathway involves the endogenous inhibition of histone deacetylases (HDAC) (Newman and Verdin, 2014). HDACs alter gene expression through the regulation of chromatin structure. HDAC2 knockout and knockdown studies in animals and cell cultures are known to increase differentiation and reduce proliferation of cancerous cells (Jurkin et al., 2011). HDAC2 knockdown is associated with upregulation of cyclin-dependent kinase inhibitors, p21 and p27, which are important enzymes in the regulation of the cell cycle (Jurkin et al., 2011). βOHB specifically seems to inhibit HDAC2 by increasing histone p21 gene expression (Mierziak et al., 2021). In hypertrophied cardiomyocytes, gene expression and metabolism are similar to fetal cells, as seen by an increased reliance on glycolytic metabolism (Razeghi et al., 2001; Fukushima et al., 2018). HDAC2 plays a role in the regulation of many fetal cardiac isoforms in cardiomyocytes, as seen in cardiac hypertrophy studies (Trivedi et al., 2007). The inhibition of HDAC2 may prevent this shift toward a Warburg-like metabolic state seen in hypertrophied cardiomyocytes (Trivedi et al., 2007). As such, βOHB may play a role in the maturation of cardiomyocytes through its regulation of HDAC2. Given the significant changes that occur in the postnatal period and the evidence regarding the KDs effect on diseased states through regulation of metabolism, ketones and their oxidation may play a role in the regulation of cell fate (Figure 2). Given the combined body of evidence, the involvement of ketones in cell maturation warrants further study.

### Glutamine Metabolism

In addition to fatty acids, carbohydrates, and ketones, amino acids are also a potential source of carbons for mitochondrial oxidative metabolism. The most prevalent of these amino acids is glutamine. Of importance, alterations in mitochondrial glutamine metabolism have been implicated in mediating cell fate. Although little is known regarding glutamine metabolism in maturing cardiomyocyte, glutamine metabolism does increase in tumor cells and is associated with an increase in cell proliferation (Kovačević, 1971; DeBerardinis and Cheng, 2009). Similar to glycolysis, glutamine metabolism seems to be favored in cancer cells due to its contribution toward anabolic metabolism. Glutamine is a nitrogen donor in the process of nucleotide biosynthesis and, as discussed previously, is key for maintaining the cellular content for the rapidly proliferating cell (Ahluwalia et al., 1990; Young and Ajami 2001). Glutamine metabolism is also a source of NADPH, which is required for anabolic processes (Vander Heiden et al., 2009). Glutamine, after being converted to glutamate, is also able to replenish the mitochondrial TCA cycle carbon pool (anaplerosis), through its deamination into α-ketoglutarate (α-KG), replenishing oxaloacetate (OAA) which provides precursors for the synthesis of nucleotides, proteins, and lipids (DeBerardinis et al., 2007; DeBerardinis et al., 2008; Ahn and Metallo, 2015). In cancerous cells, citrate is formed through glutamine-dependent reductive carboxylation, as opposed to oxidative metabolism (DeBerardinis et al., 2007; DeBerardinis et al., 2008; Ahn and Metallo, 2015). When cells are starved of glutamine, supplementation with α-KG promotes reductive metabolism, whereas OAA and pyruvate promotes oxidative metabolism (Fendt et al., 2013). α-KG produces isocitrate, citrate and acetyl-CoA, which are important for cellular biosynthesis, indicating the importance of glutamine-derived α-KG (Dyer et al., 2018). In fact, it appears that proliferating cells cannot survive without the presence of exogenous glutamine, which has been termed as a “glutamine addiction” (Eagle, 1955). *In vitro* cell lines consume ten-fold greater amounts of glutamine compared to the consumption of other amino acids (Eagle, 1955). As such, several studies have looked at inhibiting glutamine metabolism as an anti-cancer target. It has also been shown that glutamine-derived α-KG is essential for the survivability of hiPSCs (Tohyama et al., 2016). Beyond anaplerosis, glutamine is also important in the synthesis of glutathione, which, as discussed previously, is cardioprotective through its antioxidative effects (Jain et al., 2004; Wu et al., 2004; Marsboom et al., 2016). Therefore, it is possible that mitochondrial glutamine metabolism plays a role in the fate of other proliferating cells, such as the fetal cardiomyocyte, and that changes in glutamine metabolism are present in the differentiating and maturing cardiomyocyte (Figure 2).

## Summary

Changes in mitochondrial dynamics and homeostasis, as well as changes in mitochondrial energy metabolism, have a critical role in determining cell fate. Studies in fetal cardiomyocytes, cancer cells, and stem cells have provided a better understanding of how mitochondrial function and energy metabolism affect cell proliferation and differentiation. It is clear that proliferating cells rely mainly on glycolysis for energy production and its contribution to anabolic metabolism, resulting in a high Warburg effect. What is not clear is the mechanism which controls this Warburg effect, particularly in immature cardiomyocytes. In the matured/differentiated cardiomyocytes, there is a shift toward a greater reliance on fatty acid oxidation as an energy source. These changes in energy metabolism are paralleled by changes in mitochondrial dynamics and homeostasis, and a shift from increased fission and mitophagy in the proliferating state to an increase in fusion and mitochondrial biogenesis. Given the importance of changes in energy metabolism seen during this transition, the role the metabolism of other substrates, such as ketones and glutamine, have in cell fate requires further research. This understanding will be particularly important for understanding the fetal to newborn changes in the physiology and functioning of the heart, as well for applications in regenerative medicine.
